# Is Liver Enzyme Release Really Associated with Cell Necrosis Induced by Oxidant Stress?

**DOI:** 10.1155/2016/3529149

**Published:** 2015-12-20

**Authors:** Martha Lucinda Contreras-Zentella, Rolando Hernández-Muñoz

**Affiliations:** Departamento de Biología Celular y Desarrollo, Instituto de Fisiología Celular, UNAM, 04510 Mexico City, DF, Mexico

## Abstract

Hepatic diseases are a major concern worldwide. Increased specific plasma enzyme activities are considered diagnostic features for liver diseases, since enzymes are released into the blood compartment following the deterioration of the organ. Release of liver mitochondrial enzymes is considered strong evidence for hepatic necrosis, which is associated with an increased production of ROS, often leading to greater hepatic lipid peroxidation. Lipotoxic mediators and intracellular signals activated Kupffer cells, which provides evidence strongly suggesting the participation of oxidant stress in acute liver damage, inducing the progression of liver injury to chronic liver damage. Elevated transaminase activities are considered as an index marker of hepatotoxicity, linked to oxidant stress. However, a drastic increase of serum activities of liver enzyme markers ought not necessarily to reflect liver cell death. In fact, increased serum levels of cytoplasmic enzymes have readily been observed after partial hepatectomy (PH) in the regenerating liver of rats. In this regard, we are now showing that *in vitro* modifications of the oxidant status affect differentially the release of liver enzymes, indicating that this release is a strictly controlled event and not directly related to the onset of oxidant stress of the liver.

## 1. Introduction

Every organ can elicit a specific pattern of enzyme release, which remains not elucidated. Specifically, above-normal plasma enzyme activities are considered as diagnostic features for several diseases [1]. Release of enzymes usually follows their respective concentration gradients between an organ, such as the liver, and the blood compartments [2–4]. In fact, values of serum enzymes activities (“released”) are much higher than the apparent disappearance rate constants and they are also consistent with disappearance rates from plasma to lactate dehydrogenase (LDH) and aspartate (AST) and alanine (ALT) aminotransferases, after acute liver injury [5]. However, the mechanisms controlling cellular enzyme release remain poorly understood. Moreover, a drastic increase of serum activities of “liver enzyme markers” ought not necessarily to reflect liver cell death. Therefore, pathological elevations of the plasma activities of liver enzymes do not seem to be simply related to the quantitative release of such enzymes from the liver. Consequently, several enzymatic indices may be determined by differences in the time course of hepatic enzyme release, rather than reflecting true differences in the released quantities of various enzymes [5]. However, the quantitative use of enzymatic data is hampered by the fact that the fractional catabolic rate constants for the elimination of enzyme activities from plasma are unknown [5].

Release of mitochondrial enzymes from the liver is considered to provide strong evidence for hepatic necrosis [6, 7] and is also associated with specific forms of liver disease. It has been shown, for instance, that glutamate dehydrogenase (GDH) correlates well with the presence and extent of necrosis in alcoholic liver disease [8]. Furthermore, the ratio of mitochondrial and total AST (mAST) has been proposed as a marker for chronic alcoholism [9]. However, both GDH and mAST are widely distributed in various organs and lack specificity as a marker of liver injury. Despite the fact that it was reported that cumulative release of various cytosolic enzymes occurred in proportion to the corresponding activities in human control livers, the mechanisms that govern the release of liver enzymes into the bloodstream are practically unknown.

## 2. Liver Damage

Hepatic diseases are a major concern worldwide. Since the liver is a primary organ involved in biotransformation of food and drugs, hepatic disorders are very often [10]. These disorders are mainly caused by toxic chemicals, xenobiotics, and anticancer, immunosuppressant, analgesic anti-inflammatory, and antitubercular drugs [10]. Additionally, other biological agents, as well as exposure to radiations, heavy metals, mycotoxins, galactosamine, and so forth, constitute predisposing factors to develop liver damage and hepatopathy. Moreover, additional risk factors for hepatic injury include age, gender, alcoholism, and nutrition, and genetic polymorphisms of cytochrome P_450_ enzymes have also been emphasized [10]. Nutritional deficiency may predispose to drug-induced liver injury as reported in patients with HIV, tuberculosis, or alcoholism. This is largely due to the reduced hepatic glutathione in liver tissues [11]. Indeed, alcohol is believed to be one of the most important risk factors for this type of liver damage, although its exact role is not fully understood. Despite the fact that the chronic use of alcohol, particularly with malnutrition, depletes the glutathione stores, the exact link between alcoholism and liver injury is missing [12].

Chronic hepatitis B and hepatitis C are now considered to enhance the risk of drug-induced liver injury, particularly from drugs used in the treatment of tuberculosis and HIV [13]. Furthermore, a strong dose response relationship exists between drugs and hepatotoxicity. Authors further stated that drugs administered in doses of >50 mg of oral medications have an enhanced risk of this pathology [14]. In this context, the administration of nonsteroidal anti-inflammatory drugs (NSAIDs) is strongly associated with hepatotoxicity, as it is the case for nimesulide [15], diclofenac [16], and sulindac [17]. The NSAIDs, which inhibit cyclooxygenase enzymes (COX), are associated with idiosyncratic hepatotoxicity, showing symptoms ranging from elevation of serum transaminases to hepatocellular or cholestatic injury and occasionally to fatal fulminant hepatitis [18]. The mechanisms responsible for NSAID-induced liver injury may involve mitochondrial dysfunction and endoplasmic reticulum (ER) stress [19]. In addition, generation of the ER stress response induces cytochrome c (a marker of mitochondria-mediated cell death) release leading to mitochondria-mediated cell death (apoptosis). This mechanism is proposed as a major source for liver injury [20].

## 3. Liver Damage: Human Hepatic Steatosis

Liver fat deposition related to systemic insulin resistance is defined as a nonalcoholic fatty liver disease (NAFLD) which, when associated with oxidative hepatocellular damage, inflammation, and activation of fibrogenesis, can progress towards cirrhosis and hepatocellular carcinoma [21]. Due to increased onset for obesity, NAFLD is now the most frequent liver disease and the leading cause of altered liver enzymes in Western countries [21]. The NAFLD is a condition associated with obesity in which there is ectopic accumulation of triglycerides in the liver parenchyma [22]. NAFLD is used to describe a spectrum defined by liver biopsy findings ranging from accumulation of triglycerides as lipid droplets in the cytoplasm of hepatocytes, namely, simple steatosis, to the more aggressive form of nonalcoholic steatohepatitis (NASH).

Although simple steatosis appears to follow a nonprogressive course, there are still a large number of patients with NASH, some of which may also develop end-stage complications including cirrhosis [23] and hepatocellular carcinoma [24]. Considering the rapid increase in the prevalence of obesity in children globally, NAFLD is now also recognized as the most common cause of liver disease in the pediatric population [25].

As to NASH (term used in human), studies in animal models have revealed several molecular processes that recapitulate the cardinal features of NASH [24]: hepatocyte damage, inflammation, and fibrosis are the most remarkable findings in this pathology. Inflammation is a component of the wound healing process that leads to the deposition of extracellular matrix and fibrosis in the liver. A growing body of evidence supports a central role for proinflammatory cytokines, particularly tumor necrosis factor *α* (TNF-*α*) and interleukin 6 (IL-6), in the development of NASH [26].

Patients with NASH present elevated levels of TNF-*α* and IL-6 in the liver and blood, and inhibition of these cytokines has improved NAFLD in rodents [27]. A second potential mechanism is ER stress, resulting from improperly folded proteins accumulating in the ER, which elicits the unfolded protein response (UPR). The UPR activates nuclear factor *κ*B, c-Jun N-terminal kinase, and oxidative stress pathways, all of which have been implicated in progression of steatosis to NASH [28]. Studies of humans with rare inherited disorders demonstrate that hepatic TG accumulation from dietary intake, changes in the distribution of TG from adipose tissue to the liver, and/or increased de novo lipogenesis result in hepatic steatosis [29].

Hepatic steatosis is often self-limited, but it can progress to NASH (nonalcoholic steatohepatitis). NASH is distinguished from simple steatosis by the presence of hepatocyte injury (hepatocyte ballooning and cell death), an inflammatory infiltrate, and/or collagen deposition (fibrosis) [24]. It is not known whether steatosis always precedes NASH or steatosis and NASH are distinct disorders [24]. NASH, in turn, can progress to cirrhosis. In cirrhosis, hepatocytes are replaced by scared tissue composed primarily of type 1 collagen [24], produced by specialized cells called stellate cells, which are activated by liver injury and play a key role in liver regeneration. Cirrhosis can ultimately progress to liver cancer (hepatocellular carcinoma) [30]. Although obesity and insulin resistance are the most prevalent risk factors for NAFLD, hepatic fat content varies substantially among individuals with equivalent adiposity, indicating that other factors contribute to this condition [24].

In the case of alcoholic fatty liver disease (AFLD), there is an increase of NADH/NAD+ value promoted by liver alcohol oxidation, which will induce the disorder of fat metabolism, resulting in triglyceride accumulation in hepatocytes [31]. Thereby, hepatic steatosis is the early manifestation of alcohol liver disease (ALD) and is believed to be the fundamental pathological change of other more severe alcoholic liver diseases [32]. In addition, insulin pretreatment could exert a significant protective effect on oxidative damage and inflammatory reaction in the liver against ethanol exposure, but insulin can also exacerbate hepatic steatosis in mice exposed to ethanol [33]. Fatty acid synthesis in liver is mainly regulated by sterol regulatory elements binding protein-1c (SREBP-1c). Ethanol exposure can significantly activate SREBP-1c, which is responsible for the formation of fatty liver [34]. Insulin could also lead to the activation of SREBP-1c to increase the triglycerides in hepatocytes [35]. Indeed the expression of SREBP-1c can be regulated upwards by the administration of insulin and ethanol, suggesting that SREBP-1c activation might contribute to the deteriorative effects of insulin preadministration on hepatic steatosis in mice exposed to ethanol [33]. According to the “two-hit” hypothesis for ALD, even though steatosis is reversible, it might be the basis of other serious liver diseases and pathologies including steatohepatitis, fibrosis, cirrhosis, and even hepatocellular carcinoma [32].

## 4. Oxidant Stress in the Generation of Liver Damage

Overproduction of reactive oxygen species (ROS) results in oxidative stress, a state in which tissue and cellular redox balance is altered towards a more oxidizing environment [36]. ROS lead to a cumulative damage to protein, lipids, DNA, carbohydrates, and membranes. The prime functions of antioxidative defenses are suppressors of the generation of ROS, scavenging them, besides repairing and promoting reconstitution of damage, and inducing the expression of antioxidant proteins and enzymes [37, 38].

In the NAFLD, the molecular and cellular mechanisms underlying hepatic injury are not well defined. However, multiple mechanisms have been suggested, including enhanced flow of free fatty acids and release of adipocytokines from the adipose tissue [39]. In the liver, mitochondrial dysfunction, oxidative stress, and hepatocyte apoptosis are key contributors to hepatocellular injury. In addition, lipotoxic mediators and intracellular signals activate Kupffer cells, which initiate and perpetuate the inflammatory response and development of fibrosis [39]. In the development of NAFLD, there is an increased production of ROS, often leading to a greater hepatic lipid peroxidation [40, 41]. In fact, a hypercholesterolemic diet increases liver TBARS, indicating increased oxidative stress. It is known that oxidative stress can occur by increasing of prooxidant systems and/or by lowering antioxidant enzymes. Increased NADPH oxidase activity has been reported in animal models of NASH, in which dietary antioxidants or NADPH oxidase inhibitors ameliorated the progression of the disease [42, 43]. In mice, the presence of triacylglycerol and cholesterol in the diet is needed for the development of hepatic histological abnormalities of NASH and its metabolic abnormalities [44].

On the other hand, in the pathogenesis of AFLD, there is an increase in NADPH oxidase activity and predominance of prooxidant agents, exceeding the capacity of the organic antioxidant defense [45]. Under these circumstances, intracellular homeostasis in the redox status is interrupted and, sometimes, induces cell damage. This results in apoptosis or necrosis, potentially contributing to the devastating injury and dysfunction of liver tissue [46, 47]. Total body deficiency in p47^phox^ subunit of NADPH oxidase complex protects mice from alcohol-induced liver steatosis [48]. However, mice on a methionine-choline-deficient (MCD) diet develop NASH with similar pathology as the wild type, despite the lack of a functional NADPH oxidase enzyme [49]. Nevertheless, the role of this enzymatic complex in other animal models of NAFLD has not been investigated, but a role for the NADPH oxidases in chronic liver diseases related to chronic inflammation, such as fibrosis and viral hepatitis, has been proposed [50, 51].

Therefore, the present evidence strongly suggests the participation of oxidant stress in acute liver damage which, appearing to be in an accumulative effect, induces the progression of liver injury to chronic liver damage.

## 5. Effects of Vitamins and Other Antioxidants on Liver Damage

Markers for lipid peroxidation are increased in both liver and blood of patients with advanced ALD in concomitance with the lowering of antioxidant defenses [52]. Additionally supplementation with antioxidants reduced hepatic injury in alcohol-fed rodents [53].

There is evidence suggesting that the activation of AMP-activated protein kinase (AMPK) is associated with the hypoglycemic actions of metformin [54], a dimethylbiguanide, which is a commonly used antidiabetic drug [55]. Although the precise pharmacological mechanisms of metformin have not been fully elucidated, the anti-inflammatory effects of metformin involving both AMPK-dependent and AMPK-independent pathways have been mentioned [56–58]. Also, it has been suggested that metformin might have antioxidative effects both* in vivo* and* in vitro* [59–61]; metformin actually attenuates endotoxin-induced fulminant hepatitis in mice [62]. Moreover, this biguanide significantly reduces the CCl_4_-induced elevation of serum aminotransferases and hepatic histological abnormalities, which seem to be associated with decreased hepatic contents of oxidized glutathione (GSSG) and malondialdehyde (MDA) [63].

Furthermore, the Nrf2 has emerged as an indispensable regulator of both constitutive and inducible expression of detoxifying phase II and antioxidant enzyme genes in various tissues and cell types [64]. Nrf2-null mice are particularly susceptible to oxidative stress, contributing to increased hepatotoxicity by ethanol [65] and acetaminophen [66]. In rats treated with CCl_4_, there were depletion of cytoplasmic Nrf2 and suppression of Nrf2 nuclear translocation, accompanied by a dramatic downregulation of liver Nrf2 target genes, NQO1, HO-1, and GST*α*. On the other hand, increased Nrf2 expression represses the genes involved in fatty acids synthesis and, therefore, may play a crucial role in the development of NASH [67]. The activation of Nrf-2 is important for maintaining intracellular and mitochondrial GSH balance and for increasing the activities of antioxidant enzymes to protect cells from oxidative damage mediated by ethanol [68]. For instance, the treatment with *α*-lipoic acid (a vitamin) induced an early nuclear accumulation of Nrf2, resulting in a strong protection against apoptosis induced by palmitic acid [69].

Concerning vitamins, vitamin D may have a role in NAFLD pathogenesis via its effects on insulin resistance and metabolic syndrome [70]. Improvement of vitamin D status led to amelioration in serum high sensitive-CRP and MDA in patients with NAFLD. Therefore, vitamin D could be considered as an adjunctive therapy to attenuate systemic inflammation and lipid peroxidation along with other treatments administered to patients with NAFLD [71]. It is also known that another vitamin, vitamin E, a potent antioxidant that protects against oxidative stress induced liver damage* in vitro* and* in vivo*, has beneficial effects on histological outcomes in patients with NAFLD. This vitamin decreases serum levels for ALT activity in patients with HCV genotype 3, suggesting that vitamin E has a protective effect against HCV-induced liver cells necrosis [72].

In the same way, the diallyl disulfide, primarily derived from the garlic, effectively ameliorates CCl_4_-induced oxidative hepatic injury and inflammatory responses in rats [64]. The hepatoprotective effects of diallyl disulfide may be due to its ability to induce antioxidant or detoxifying enzyme activities through activation of Nrf2 and to suppress the production of inflammatory mediators by inhibiting NF-*κ*B activation. These properties confer to this molecule a useful protective effect against various hepatic injuries caused by oxidative stress and inflammatory response [64].

The effects of diverse antioxidants protecting or ameliorating liver injury also emphasize the important role of oxidant stress in the generation of acute and chronic liver damage.

## 6. Serum (“Marker”) Enzyme Activities and Liver Damage

Several hepatotoxins such as chemicals, drugs, lipopolysaccharides, heavy metals, and mycotoxins elicit a wide variety of hepatic injuries. Numerous enzymes are produced in the liver and are normally distributed within the cells of the liver [10]. Elevation of serum enzyme is taken as the sensitive biomarker of liver toxicity. The determination of various liver enzymes in serum, as ALT, AST, alkaline phosphatase (ALP), *γ*-glutamyl transpeptidase (*γ*-GGTP), lactate dehydrogenase (LDL) in serum, and serum lipid profile, cholesterol, triacylglycerides, and lipoproteins, are used to evaluate the functional status of the liver and to detect liver injury. An elevation in transaminase in conjunction with a rise in bilirubin level to more than double is considered as a marker index of hepatotoxicity [73]. Other sensitive biomarkers of liver function are albumin concentration, total protein (TP), and prothrombin time (PT). These biomarkers can serve as an index of liver biosynthetic capacity [10]. Therefore, ALT and AST activities in serum are the most frequently used indicators for evaluation of liver injury [74], meeting drastic increases under these conditions [75]. The levels of cholesterol were similar in patients control and with NAFLD, but those with NAFLD had higher triglyceride levels [21]. A hypercholesterolemic diet causes liver damage and increased oxidative stress and cholesterol levels in female rats. The resultant liver injury was characterized by hepatomegaly and accompanied by increased activities of AST and ALT enzymes [76]. Even more, a large proportion of patients with chronic inflammatory liver diseases and of patients with metabolic syndrome complications had impaired glucose tolerance [70].

Alcoholic subjects having moderate/severe hepatic steatosis usually present an increase in the levels of triglycerides, cholesterol, glucose, *γ*-GGTP, ALT, bilirubin, *α*-1 and *β*-2 globulins, and iron and a decrease in the levels of AST [77]. In this regard, it has been found that in alcoholic subjects the AST/ALT ratio is significantly increased and it has been considered that the AST/ALT ratio could be a marker playing a role for alcoholic liver disease progression [77].

However, there exist discrepancies when matching changes of assumed “liver enzymes” in serum and other markers for liver integrity. For instance, increased levels of *γ*-GGTP, a liver enzyme, play an independent role in the pathogenesis and clinical evolution of cardiovascular disease induced by atherosclerosis and are associated with increased cardiovascular disease mortality [78]. Moreover, other authors reported similar results in the association of high *γ*-GGTP levels with fatal and nonfatal cardiovascular disease, independently of the metabolic risk factors and alcohol consumption [78]. On the other hand, some studies also reported the association of increased ALT levels and cardiovascular disease. Moreover, between patients without viral hepatitis or excessive alcohol consumption, those with elevated ALT level had a higher calculated risk of cardiovascular disease than those with normal ALT activity [78].

Therefore, several situations arise where there is evident loss of correlation between serum levels of liver enzymes and tissue necrosis and in the specificity of possible tissue markers.

## 7. Fluctuations of Serum “Marker” Enzymes in the Model of Liver Regeneration Induced by Partial Hepatectomy in Rats

In clinical practice, net enzyme release could be indicative of liver damage, even though hepatic enzyme activities can remain normal [79] or even elevated in the organ [80]. In CCl_4_-induced hepatic injury, serum and liver enzyme activities vary according to the enzyme studied [81], but frequently the appearance of mitochondrial enzymes in the serum is delayed as compared to the cytoplasmic enzymes [80, 82]. MDH and AST activities found in perfusates from isolated livers are mainly derived from their cytoplasmic isozymes [2, 80].

Hence, enzyme release might depend on alterations in plasma membranes, mitochondrial dysfunction, and/or changes in cellular volume regulation [1, 83, 84]. In addition, the level of increased serum enzyme activities would also depend on the susceptibility of the liver cell type being damaged [85, 86].

However, a discrepancy exists between a remarkable increase in serum enzyme activities and structural and functional characteristics found after hepatic resection. Remaining hepatocytes can restore the original mass of the organ, through a process widely known as liver regeneration [87]. Partial hepatectomy- (PH-) induced liver regeneration and enzyme release have been described in detail in the literature over the past 30 to 40 years. From this information, augmented levels of serum transaminases have been found in rats subjected to PH [88], while increased serum activity of ornithine carbamoyltransferase (OCT), a liver mitochondrial enzyme, was also found after PH [89]. Similarly, patients subjected to partial removal of the organ showed a “selective” release of liver enzymes, with the serum activity of OCT being the most enhanced in these patients [90]. Despite the regenerative capacity of the remnant liver, and independently of the extent of the liver resection, increased serum levels of cytoplasmic enzymes have readily been observed after PH in rats [91]. Increases in serum levels of liver enzymes were greater and more prolonged after 85% PH, which is accompanied by a marked mortality rate in rats suffering the largest liver mass loss [91].

While the latter has been interpreted as a consequence of progressive necrosis and liver failure after massive PH, in other models of liver injury and regeneration, increased serum ALT and AST did not correlate with cell necrosis. For example, liver injury and regeneration induced by acute carbon tetrachloride administration to rats occur irrespective of the extent of the increase in serum activities for these aminotransferases [83]. These findings support the suggestion that enhanced serum enzymes could be distinctly separable from prior elevations induced by tissue damage produced by carbon tetrachloride [81]. Therefore, the reason for a substantial increase in serum activities for liver enzymes is controversial in the case of PH-induced liver regeneration in rats.

A drastic increase of serum activities of “liver enzyme markers” does not necessarily have to reflect liver cell death. Indeed, we demonstrated recently that an important fraction of the released hepatic enzymes depends largely on hemodynamic changes in the rat liver [92].

Taking advantage of the model of two-thirds partial hepatectomy- (PH-) induced rat liver regeneration (“small-for-size liver”), we showed that liver cell proliferation occurs accompanied by a selective PH-induced elevation of serum enzymes, not related to hepatocellular necrosis [93] nor to mitochondrial dysfunction [94]. Indeed, the PH induction of specific enzymes (predominantly those from mitochondria) is partly regulated by flow-bearing physical forces and is independent of extrahepatic factors [92]. Similarly, patients subjected to partial removal of the organ, who were candidates for liver transplantation, showed a “selective” release of liver enzymes, where serum activity for OCT was the most enhanced [92]. Currently, it is known that mechanical forces can be converted into a sequence of intracellular biochemical signals targeting cells, as it occurs in the endothelial layer [95]. Hence, the physicochemical interactions within cells have become a fascinating field in the study of cell functioning, and the release of enzymes by vascular organs might constitute another event regulated by hemodynamic forces.

A number of intracellular events triggered by fluid shear stress have been elucidated and mechanisms causing these events have been proposed [96]. These include direct stimulation of luminal surface transmembrane proteins, activation of ion channels affecting intracellular Ca^++^[Ca^2+^]_i_ mobilization [97] which has been postulated as a likely regulator of cell proliferation [98, 99], and production of nitric oxide (NO) [100]. These mechanisms allow the transduction of stress along cytoskeletal elements to other regions of the cell. Changes in the endothelial cell membrane may act as primary mechanoreceptors in response to shear stress. We have recently suggested a possible role for cell-mediated mechanotransduction in liver enzyme release mediated by increasing shear stress, which selectively affected the release of liver enzymes. Therefore, we demonstrated that flow-induced shear stress can control the amount of hepatic enzymes released into the bloodstream, which is largely regulated through modifications in cell calcium mobilization and production of liver NO. These events were markedly elevated in the proliferating rat liver [101].

## 8. The Effect of Pro- and Antioxidant Environments on* In Vitro* Liver Enzyme Release

The liver is capable of recovering from damage or loss of up to 90% of its mass by means of proliferative activity, restoring it to normal size. This process, known as liver regeneration, involves the endocrine and paracrine actions of growth factors and the activation of specific protooncogenes and of transcription factors [102]. However, the understanding of the delicate coordination that triggers, modulates, and stops this process is still not well understood.

The experimental model of cell proliferation and growth regulation was examined regarding the production of free radicals and the rate of lipid peroxidation (LP). The model showed that LP, promoted by PH and CCl_4_ administration, is qualitatively distinct among subcellular fractions and may indeed be a normal cell event of physiological importance in the regenerating liver. Thus, LP plays a role in the early steps of liver regeneration [103].

### 8.1. Release of Liver Enzymes by Liver Slices

We recently made experiments to assess the* in vitro* impact of pro- and antioxidant conditions on enzyme release from control and regenerating rat livers. We used male Wistar rats (230–280 g of body weight) fed* ad libitum* and maintained under a 12 h light/dark period, which were subjected to two-thirds PH, while sham-operated (laparotomy) animals provided a control for surgical conditions [103]. Twenty hours after surgery, liver slices were obtained and incubated under basal (B) conditions described by Díaz-Juárez et al. [92]. Then, the oxidant status of the liver slices was changed by adding 400 *μ*mol/L hydrogen peroxide (H_2_O_2_), as prooxidant, or 400 *μ*mol/L butylated hydroxytoluene (BHT), as antioxidant. The liver slices were incubated under basal conditions in sealed flasks at 37°C for one hour in the presence of 5 mmol/L glucose and after 15 min of oxygenation with a 95% O_2_ : 5% CO_2_ mixture.

**Figure 1:**
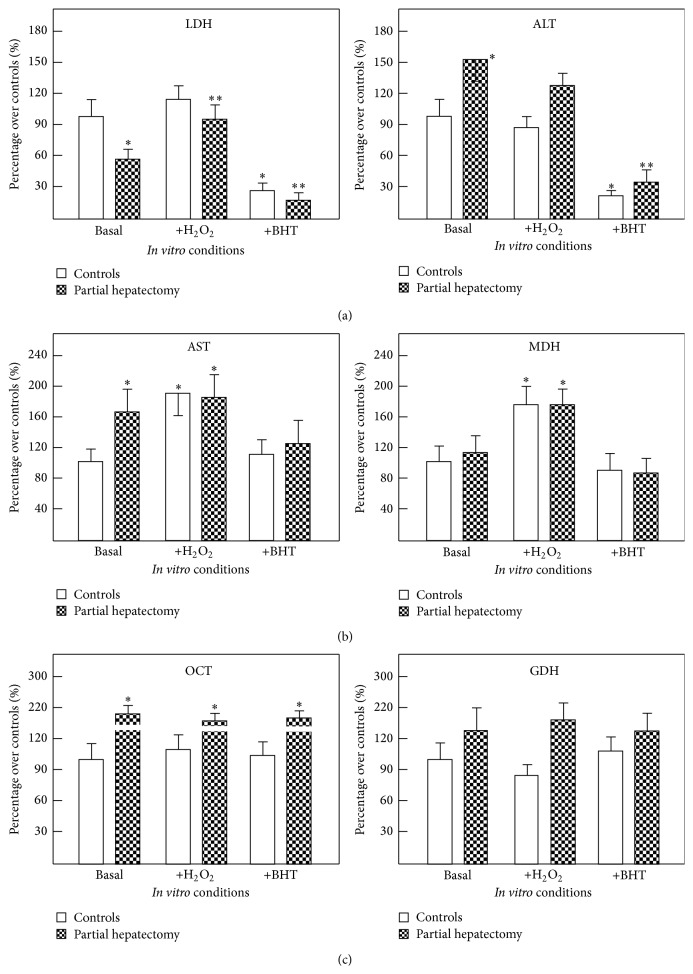
Effects of changing the oxidative status on cytoplasmic and mitochondrial enzymes from liver slices from control and PH rats. In panel (a), results of released ALT and LDH (cytoplasmic enzymes) are expressed as mean ± SE of six individual observations per experimental point. In panel (b), results of release of MDH and AST (enzymes sharing cytoplasmic and mitochondrial localization) are expressed and, in panel (c), those of the release of OCT and GDH activities (mitochondrial enzymes) are expressed. Enzyme release was tested under basal conditions. Abbreviations for the compounds used: hydrogen peroxide (H_2_O_2_) and butylated hydroxytoluene (BHT). Statistical significance: ^*∗*^ *p* < 0.01 against the group of sham-operated controls (basal conditions); ^*∗∗*^ *p* < 0.01 versus PH rats group (basal conditions).

As shown in Figure 1, we found that LDH and ALT (cytoplasmic enzymes) were influenced by the oxidant status. In control (sham-operated) rats, LDH and ALT release was significantly diminished by the addition of the BHT antioxidant. Liver slices from PH rats released significantly more ALT into the incubation medium, which was also inhibited by BHT. Additionally, the regenerating liver had a lower LDH release, which was increased by prooxidant conditions given by the added hydrogen peroxide (Figure 1(a)). As to AST and malate dehydrogenase (MDH) (sharing cytoplasmic and mitochondrial compartments) release was increased under prooxidant conditions in liver slices from both control and hepatectomized rats (Figure 1(b)). The release for the mitochondrial enzymes, OCT and GDH, was unaffected by the use of pro- and antioxidants agents (Figure 1(c)).

The results indicate that modifications of the oxidant status affected differentially the enzymes tested, cytoplasmic, mitochondrial, or sharing cytoplasmic and mitochondrial compartments. Therefore, the data obtained through the experiments would suggest that release of hepatic enzymes is a strictly controlled event, which is not linearly related to the changes in the oxidant status of the liver.

### 8.2. Release of Enzymes by Isolated Liver Mitochondria

Although 70% PH in rats induces the release of mitochondrial matrix proteins into the cytosol [104], liver mitochondrial function is efficiently preserved [94] and necrotic or apoptotic events have not been conclusively found in the rat regenerating liver [105].

**Figure 2:**
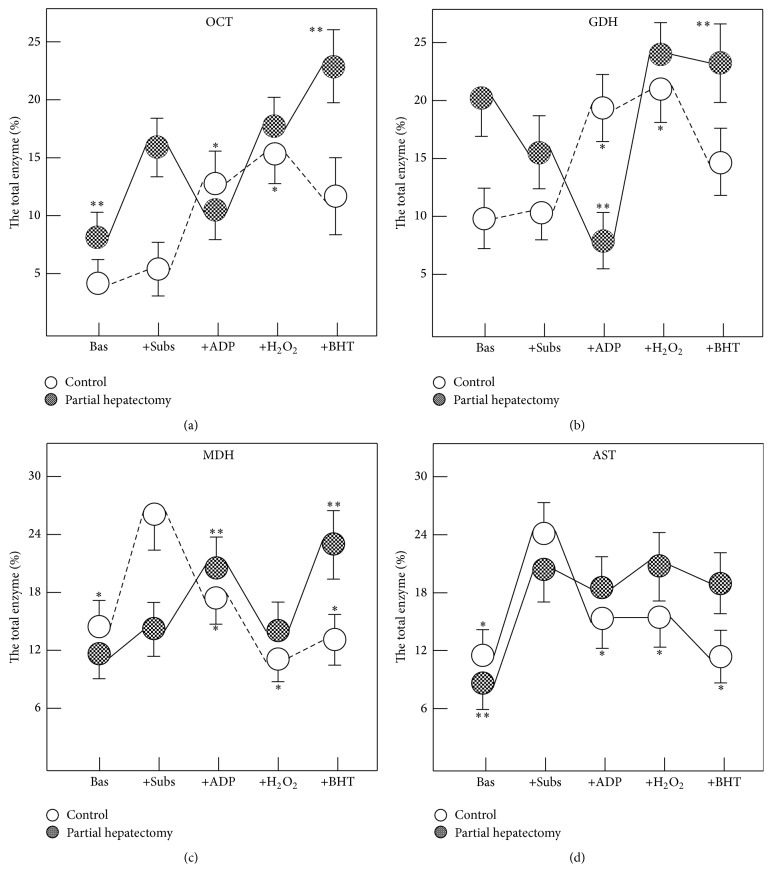
Effects of changing the oxidative status on enzyme release from isolated liver mitochondria from control and PH rats. Results are expressed as mean ± SE of four individual observations per experimental point and correspond to the percentage of the released enzyme in respect to the total mitochondrial activity for each enzyme. Bas: basal, Subs: substrates, H_2_O_2_: hydrogen peroxide, and BHT: butylated hydroxytoluene. Statistical significance: ^*∗*^ *p* < 0.01 against the group of sham-operated controls (incubated with substrates); ^*∗∗*^ *p* < 0.01 versus PH rats group (incubated with substrates).

To study the release of enzymes from isolated mitochondria, a mitochondria pellet was obtained by differential centrifugation from livers obtained from control and PH rats (24 hs after treatments). Mitochondrial respiration and phosphorylation were measured as previously described in detail [94]. When incubated in a protein-free medium in the absence of substrates, isolated liver mitochondria were able to release enzymes contained at the mitochondrial matrix. The maximal release was reached during the first 15 min at 37°C (Figure 2). In control preparations, the release of OCT, a mitochondrial enzyme, was not affected by addition of substrates for the electron transport chain (glutamate-malate and succinate), but addition of ADP (phosphorylating condition) enhanced OCT release. Under phosphorylating conditions, prooxidant (with hydrogen peroxide) or antioxidant (BHT) environments had no significant effects on OCT release. On the contrary, isolated liver mitochondria from PH rats, incubated with the substrates, greatly increased the OCT release, whereas the addition of ADP returned the release of the enzyme to the basal condition (Figure 2(a)). There are significant differences in the release of OCT between control and mitochondrial preparations from PH rats in the prooxidant condition. In this condition more OCT was released and this effect was surprisingly more accentuated in the presence of BHT (Figure 2(a)). As in OCT, the addition of substrates plus ADP also elicited an opposite profile in the mitochondrial GDH release; the comparison between control and HP rats showed that, in controls, GDH release was significantly increased under phosphorylating conditions, while in PH rats GDH release was significantly inhibited (Figure 2(b)). Whereas changes in the oxidant status did not affect GDH release in control mitochondria, a significant increase of GDH release was noted after addition of either hydrogen peroxide or BHT to isolated mitochondria from PH-animals (Figure 2(b)). The MDH release (as AST, localized both in cytosol and in the mitochondria), under phosphorylating conditions, followed a distinct pattern: in control preparations, incubation with substrates enhanced MDH release, whereas under phosphorylating conditions, this release returned to that found in the basal conditions. In mitochondria from PH rats, neither addition of substrates nor addition of ADP had a significant effect on MDH release (Figure 2(c)). Moreover, changes in the oxidant status did not have significant effects on MDH release in mitochondrial preparations from either control or PH-animals (Figure 2(c)). Finally, release of AST from isolated mitochondria from both experimental groups was increased only after the addition of substrates (Figure 2(d)). Both, the prooxidant condition and the addition of BHT significantly reduced the AST release only in mitochondrial preparations isolated from control animals (Figure 2(d)). We observed again that the oxidant status affects in a differential manner the release of liver mitochondria enzymes and that there was no constant pattern of changes in this parameter in function of fluctuations imposed* in vitro* in the oxidant status.

## 9. Conclusions

The liver is a primary organ involved in biotransformation of food and drugs. Moreover, the increased specific enzyme activities in the blood are considered as diagnostic features for liver diseases. However, a drastic increase of serum activities of liver enzyme markers ought not necessarily to reflect liver cell death. Release of mitochondrial enzymes from the liver is considered to provide strong evidence for hepatic necrosis and also is associated with specific forms of liver disease.

It has been frequently reported that in the development of liver diseases there is an increased production of ROS, often leading to greater hepatic lipid peroxidation. Lipotoxic mediators and intracellular signals activate Kupffer cells, which initiate and perpetuate the inflammatory response and development of fibrosis. This evidence strongly suggests the participation of oxidant stress in acute liver damage, probably inducing the progression of liver injury to chronic liver damage. It is known that elevated transaminase activities in conjunction with a rise in bilirubin level to more than double are considered as a marker index of hepatotoxicity, linked to oxidant stress. However, there exist discrepancies when matching changes of assumed “liver enzymes” in serum and other markers for liver integrity. In fact, there are several situations where an evident lack of correlation exists between serum levels of liver enzymes and tissue necrosis and in specificity as tissue marker. Despite the regenerative capacity of the remnant liver after PH, and independently of the extent of liver resection, increased serum levels of cytoplasmic enzymes have readily been observed after PH in rats. Similarly, patients subjected to partial removal of the organ showed a “selective” release of liver enzymes, serum activity of OCT being the most enhanced in these patients. Taking advantage of the model of PH-induced rat liver regeneration (“small-for-size liver”), we showed that liver cell proliferation occurs accompanied by a selective PH-induced elevation of serum enzymes. Here, we additionally demonstrated that* in vitro* modifications of the oxidant status differentially affected the enzymes tested in our laboratory in cytoplasmic, mitochondrial, or sharing cytoplasmic and mitochondrial compartments. Therefore, the data obtained would suggest that the release of hepatic enzymes is an event strictly controlled and not directly related to the onset of oxidant stress of the liver.
